# Pharmacologic down-regulation of EZH2 suppresses bladder cancer *in vitro* and *in vivo*

**DOI:** 10.18632/oncotarget.1867

**Published:** 2014-03-26

**Authors:** Shou-Hung Tang, Hsu-Shan Huang, Hong-Ui Wu, Yi-Ta Tsai, Mei-Jen Chuang, Cheng-Ping Yu, Shih-Ming Huang, Guang-Huan Sun, Sun-Yran Chang, Pei-Wen Hsiao, Dah-Shyong Yu, Tai-Lung Cha

**Affiliations:** ^1^ Division of Urology, Department of Surgery, Tri-Service General Hospital, National Defense Medical Center, Taipei, Taiwan, R.O.C.; ^2^ College of Medical Science and Technology, Taipei Medical University, Taipei, Taiwan, R.O.C.; ^3^ Graduate Institute of Life Sciences, National Defense Medical Center, Taipei, Taiwan, R.O.C.; ^4^ Graduate School of Biomedical Science, National Defense Medical Center, Taipei, Taiwan, R.O.C.; ^5^ Department of Pathology, Tri-Service General Hospital, National Defense Medical Center, Taipei, Taiwan, R.O.C.; ^6^ Department of Biochemistry, National Defense Medical Center, Taipei, Taiwan, R.O.C.; ^7^ Taipei City Hospital, Taipei, Taiwan, R.O.C.; ^8^ Agricultural Biotechnology Research Center, Academia Sinica, Taipei, Taiwan, R.O.C.; ^9^ Department of Immunology, National Defense Medical Center, Taipei, Taiwan, R.O.C.

**Keywords:** EZH2, NSC745885, proteasome degradation, G2/M cell-cycle arrest

## Abstract

The polycomb group gene, EZH2, is highly expressed in advanced bladder cancer. Here we demonstrated that down-regulation of EZH2 in tumor tissues after neo-adjuvant chemotherapy correlated with good therapeutic response in advanced bladder cancer. We next developed a small molecule, NSC745885, derived from natural anthraquinone emodin, which down-regulated EZH2 via proteasome-mediated degradation. NSC745885 showed potent selective toxicity against multiple cancer cell lines but not normal cells. NSC745885 treatment overcame multiple-drug resistance and inhibited growth of resistant cancer cells. Over-expression of EZH2 in cancer cells attenuated effects of NSC745885, suggesting that down-regulation of EZH2 was responsible for growth inhibition of NSC745885. NSC745885 also suppressed tumor growth and down-regulated EZH2 *in vivo*. These results indicate that NSC7455889 suppresses bladder cancer by targeting EZH2.

## INTRODUCTION

Over the past decade, more than 350 new agents have reported to be in clinical development for cancer and cancer-related indications. Unfortunately, the pace of approval for newly-developed agents has slowed down since 2000 [1-3]. The approval process is often burdened by many difficulties, which can be divided into two categories: i) the safety profile and therapeutic efficacy of the new agent to reflect its survival benefits, and ii) the failure to identify essential drug target(s) responsible for cancer biology [1]. For a new agent to be translated for clinical trial, it must have selective toxicity with specific target(s) for cancer cells rather than normal cells.

Historically, most successful new drugs are from either natural products or compounds derived from natural products. Doxorubicin, mitomycin-C, and docetaxol are from natural products and have revolutionized medicines for clinical cancer treatment. However, natural product research has been significantly reduced of late. A major obstacle is the difficulty in discovering naturally-produced drug candidates with potent, anti-cancer biological activity. Currently, the favored method is high throughput screen (HTS) of massive libraries of pure synthetic compounds [4], but the output has been quite low, with <0.001% hit rate (HR), compared to 0.3% HR for natural products [5].

Another critical factor in new anti-cancer drug development is the identification of crucial drug target(s) responsible for cancer biology. The identification and characterization of growth-factor and angiogenesis signaling pathways that are crucial to the establishment, progression, and metastases of tumors have brought a new paradigm in cancer treatment: “targeted or personalized therapy”. Anti-epidermoid growth factor receptor (EGFR), imatinib, anti-HER2, heceptin, anti-angiogenesis, and sunitinib are targeted therapies widely used in the clinics. Yet despite encouraging clinical outcomes, they rarely achieve comprehensive success. Thus, it is still imperative to elucidate real fundamental factor(s) that cause aggressive malignant behaviors as drug target(s).

The enhancer of zeste homolog 2 (EZH2) is a member of the polycomb repressive complex 2 (PRC2). With its major partners EED and SUZ12, it catalyzes the di- and tri-methylation of histone H3 lysine 27 [6-9] but unlike EED and SUZ12, it is essential for both embryonic stem cell pluripotency and self-renewal [10-12]. Moreover, EZH2 expression is up-regulated in various cancers [13-16] and has been found to serve as a marker of the aggressive stages and clinical behavior of bladder and prostate cancers [17, 18]. The ectopic over-expression of EZH2 contributes to the malignant transformation of normal prostatic cells [19, 20] and hyperplasia in breast epithelium [21, 22], while its post-translational modification alters oncogenic effects. Tumorigenicity is increased when it is phosphorylated by Akt at Ser 27, which attenuates its methyltransferase activity [23]. Wei et al. have revealed that the *phosphorylation* of Thr 487 by CDK1 attenuates it methyltransferase activity resulting in inhibition of cancer cell invasion [24, 25]. All these oncogenic effects of EZH2 on cancer cells make it a perfect target of new anti-cancer drug development.

Emodin (1,3,8-trihydroxy-6-methylan-thraquinone) is an active constituent extracted of the rhizome of Rheum palmatum. Literature suggests that emodin possesses anti-bacterial [26], anti-inflammatory [27], immuno-suppressive [28], and anti-cancer effects [29]. It also down-regulates androgen receptors and suppresses prostate cancer cell growth [30]. It has been previously shown to modulate the EZH2-mediated H3 K27 trimethylation of bladder cancer cells [31]. The multiple effects of naturally-produced emodin are intriguing such that it has been used as a candidate for anti-cancer drug development by synthesizing its derivatives.

In the present study, the cell-based platform with GFP-EZH2 expression cancer cells is used to screen multiple emodin derivatives that can target EZH2 for new drug identification. The results show that an emodin derivative, NSC745889, has potent anti-cancer effects by down-regulating EZH2 through a proteasome-mediated degradation pathway. Furthermore, NSC745889 has selective cytotoxicity towards cancer cells but not normal cells, can cause G2/M cell cycle arrest, and can overcome multi-drug resistance to inhibit cancer cell growth *in vitro* and suppress tumor growth *in vivo*. These results indicate that NSC7455889 may be a potential anti-cancer medicine by targeting oncogenic EZH2.

## RESULTS

### Down-regulation of EZH2 expression correlated with treatment response to neo-adjuvant chemotherapy

**Figure 1:**
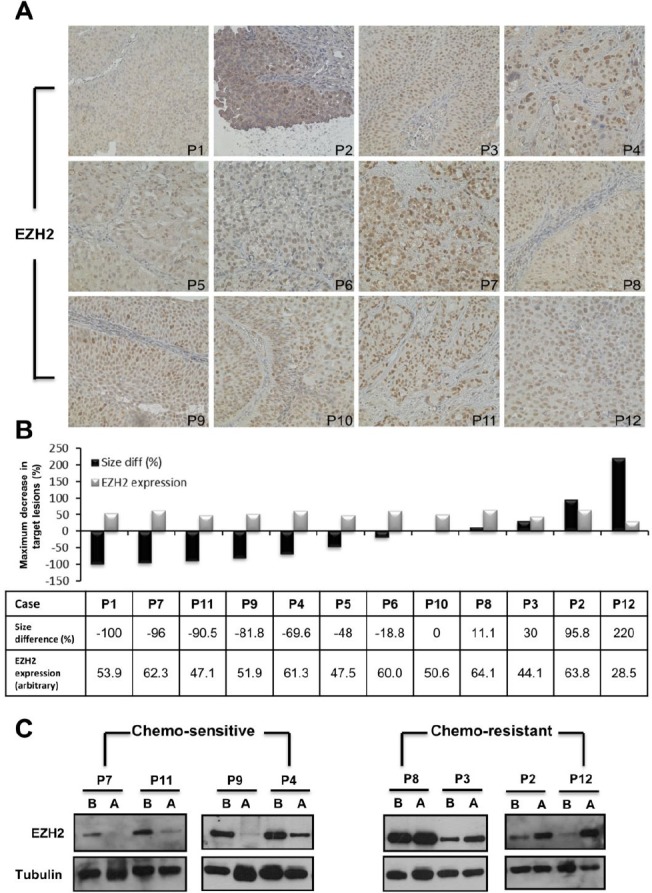
Down-regulation of EZH2 in bladder cancer tissues correlated with the therapeutic efficacy of neo-adjuvant chemotherapy (A) EZH2 levels were analyzed in 12 bladder cancer cases by immuno-histochemistry. (B) A representative graph showed the relative intensities of EZH2 among bladder tumor tissues (grey bars) and the relative change in tumor volume after six courses of neo-adjuvant chemotherapy of patients with advanced bladder cancers (black bars). C. EZH2 expression was assayed in four pairs of chemo-sensitive and chemo-resistant bladder cancers by Western blotting. B, before chemotherapy; A, after chemotherapy

To analyze the EZH2 expression in bladder cancer tissues from 12 patients who underwent neo-adjuvant chemotherapy, immuno-histochemical analysis of archived human paraffin-embedded bladder urothelial carcinoma were selected (Supplemental Table 1). Staining serial sections of tumor samples with EZH2 revealed that EZH2 was highly expressed in 12 bladder cancer tissues (Fig. 1A). However, the baseline EZH2 expression levels in bladder cancer tissues did not correlate with the therapeutic response to neo-adjuvant chemotherapy among the 12 patients (Fig. B).

Eight pairs of fresh frozen cancer tissues were obtained from patients before and after neo-adjuvant chemotherapy. Four patients with good therapeutic response had significantly down-regulated EZH2 expression in bladder cancer tissues after neo-adjuvant chemotherapy. In contrast, unchanged or enhanced EZH2 expression after neo-adjuvant chemotherapy correlated with poor therapeutic response (Fig. 1C). Thus, EZH2 might play a role in mediating drug resistance to chemotherapy.

### NSC745885 selectively inhibited cancer cell growth and overcame drug resistance

**Figure 2:**
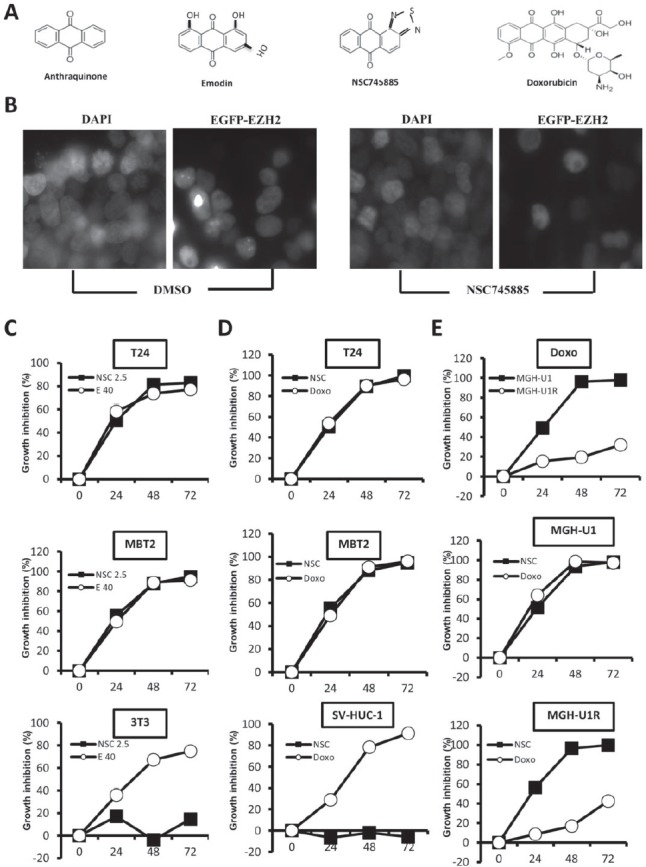
NSC745885 selectively inhibited cancer cell growth and overcame drug resistance compared to emodin and doxorubicin (A) NSC745885 shared a core structure with anthraquinone category drugs like emodin and doxorubicin. (B) The GFP-EZH2 expression of 293T cells treated with DMSO or 5 μM NSC745885 was detected by microscopy. (C) Bladder cancer cells, T24, MBT2, and normal fibroblast 3T3 cells were treated with NSC745885 (2.5 μM) and emodin (40 μM) for 24, 48, and 72 h, respectively. Growth inhibition after NSC745885 treatment compared to emodin treatment was determined by MTT assay (n=3). (D) Growth inhibition by NSC745885 treatment on T24, MBT2, and immortalized normal urothelial cells SV-HUC-1 compared to doxorubicin (2.5 μM) treatment was determined by MTT assay (n=3). (E) Growth inhibition by NSC745885 treatment on MGH-U1 and MGH-U1R cells compared to doxorubicin treatment was determined by MTT assay (n=3). All values were mean±SEM.

A synthetic compound NSC745885 was generated from emodin. NSC745885 shared a core structure with anthraquinone category drugs like doxorubicin (Fig. 2A). Using a cell-based platform with GFP-EZH2 expressing 293T cells, NSC745885 was identified as having potent activity to specifically down-regulate EZH2 but not EZH1 (Fig. 2B and Supplemental Fig. 1). Its growth inhibition effect was first tested to determine its IC_50_ on MBT-2, T24 bladder cancer cells, and SV-HUC-1 immortalized normal urothelial cells by titrating different dosages in different time periods (Supplemental Fig. 2).

The growth inhibition effect of NSC745885 was compared to emodin and doxorubicin and MTT assay was used to measure cell viability at indicated times. Results demonstrated that 2.5 μM NSC745885 achieved the same growth-inhibitory effect as 40 μM emodin and 2.5 μM doxorubicin on two bladder cancer cell lines, MBT2 and T24. However, NSC745885 was less toxic to normal 3T3 fibroblast and SV-HUC-1 immortalized normal urothelial cells compared to emodin and doxorubicin (Figs. 2C and 2D).

The growth-inhibitory effect of NSC745885 was further compared to doxorubicin on one pair of bladder cancer cell line MGH-U1 and its derivative MGH-U1R with multi-drug resistance. NSC745885 and doxorubicin showed the same potency of growth inhibition on MGH-U1 cells. As expected, MGH-U1R possessed up to 40-fold resistance to doxorubicin compared to its parental MGH-U1 cells. Interestingly, the same dosage of 2.5 μM NSC745885 treatment resulted in complete suppression of MGH-U1R cell growth as observed in MGH-U1 cells (Fig. 2E).

### NSC745885 down-regulated EZH2 and induced G2/M cell-cycle arrest of cancer cells

**Figure 3:**
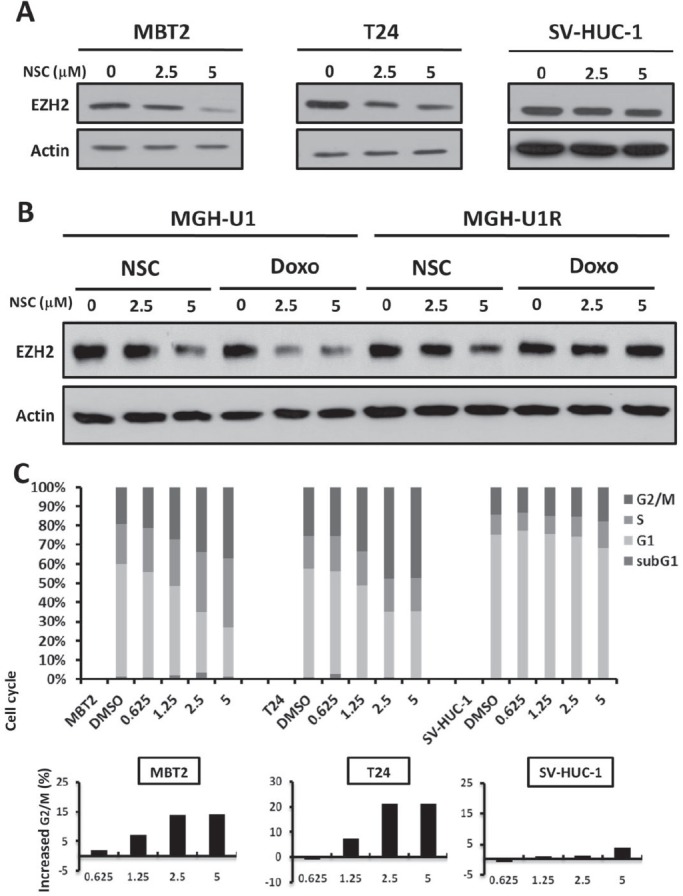
NSC745885 treatment resulted in the down-regulation of EZH2 and G2/M cell-cycle arrest in bladder cancer cells (A) T24, MBT2, and SV-HUC-1 cells were treated with the indicated amount of NSC745885 for 24 h. (B) MGH-U1 and MGHUR cells were treated with the indicated amounts of NSC745885 and doxorubicin for 24 h. Whole cell lysates were harvested and prepared for Western blotting with the indicated antibodies. (C) The cells were treated with indicated dosages of NSC745885 and DMSO control (labeled as DMSO) for 24 h. The cell cycle was analyzed by FACS analysis (mean±SEM, n=3). The percentage of cells in the defined stage of cell cycle was determined by calculating DNA content histograms. The increased G2/M proportion was plotted in the barograph. Black, sub-G1 phase; white, G1 phase; light gray, S phase; dark gray, G2/M phase

The EZH2 expression levels in the aforementioned cell lines were further checked before and after NSC745885 treatment. NSC745885 down-regulated EZH2 in MBT2 and T24 bladder cancer cells with high baseline EZH2 expression levels, but not in SV-HUC-1 immortalized normal urothelial cells (Fig. 3A). Moreover, NSC745885 and doxorubicin both decreased EZH2 protein levels in drug-sensitive MGH-U1 bladder cancer cells, but only NSC745885, not doxorubicin, down-regulated EZH2 expression in multi-drug-resistant MGH-U1R bladder cancer cells (Fig. 3B).

To further address the question why NSC745885 selectively down-regulated EZH2 in bladder cancer cells than in normal urothelial cells, the phosphorylation status at serine 21 and threonine 487 of EZH2 was examined in MBT2, T24, and SV-HUC-1 cells. There were no different basal levels of S21 and T487 phosphorylation of EZH2 among MBT2, T24 and SV-HUC-1 cells. The wild-type or mutant EZH2 (21A, 487A, mimicking non-phosphorylation, and 21D, 487D, mimicking phosphorylation) were transiently transfected into 293T cells. NSC745885 treatment resulted in equal down-regulation of wild-type and mutant EZH2 in 293T cells (Supplemental Fig. 3).

The cell cycle profiles of these bladder cancer cell lines and immortalized normal bladder urothelial cells were further investigated subsequent to NSC745885 treatments, with DMSO-treated cells as controls. NSC745885 treatment induced G2-M cell cycle arrest in MBT2 and T24 bladder cancer cells, but not in SV-HUC-1 cells, in a dose-dependent manner for 24 h (Fig. 3C, upper panel). NSC745885 treatment increased up to 15% and 20% of MBT2 and T24 cells, respectively, in the G2/M phase (Fig. 3C, lower panel). NSC745885 treatment also had no significant change in cell cycle distribution of SV-HUC-1 immortalized normal urothelial cells, consistent with less toxic results by MTT assays.

Furthermore, the main proteins responsible for G2/M cell cycle transition, such as Aurora A, B, cdc2, and Cdc25C, were down-regulated under NSC745885 treatment in MBT2 and T24 bladder cancer cells, but not in SV-HUC-1 immortalized normal urothelial cells (Supplemental Fig. 4)

### NSC745885 down-regulated EZH2 and re-expressed its downstream-silenced tumor suppressor genes

**Figure 4:**
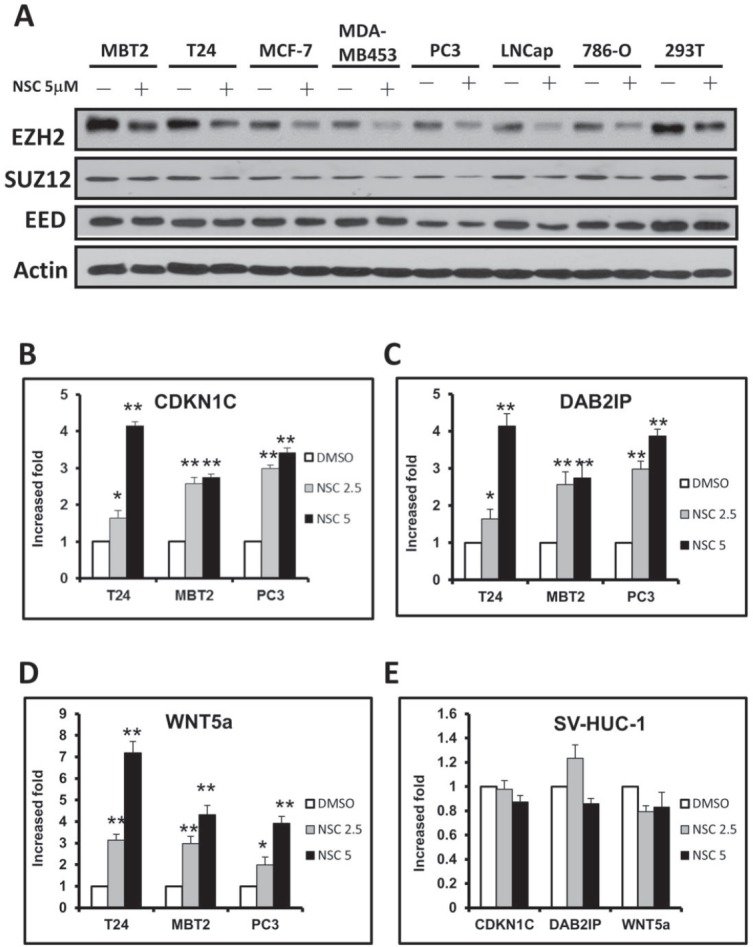
NSC745885 treatment resulted in the down-regulation of EZH2 and up-regulation of EZH2-silencing tumor suppressor genes in multiple cancer cell lines (A) Multiple cancer cell lines (as indicated) were treated with 5 μM NSC745885 for 24 h. Whole cell lysates were harvested and prepared for Western blotting with the indicated antibodies. (B-D) T24, MBT2, PC3, and SV-HUC-1 cells were treated with 2.5 and 5 μM NSC745885 or DMSO for 24 h. Relative mRNA expressions were verified by quantitative real-time PCR. The expression levels of CDKN1C, DAB2IP and WNT5a were normalized to GADPH expression relative to DMSO control and expressed as “relative expression” (mean±SEM, n=3).

The effects of NSC745885 on EZH2 expression levels in different cancer cell lines were further investigated. NSC745885 treatment down-regulated the EZH2 expression in eight cancer cell lines, including prostate, breast, kidney, and bladder cancers. However, the major components of polycomb repressive complex 2, SUZ12 and EED, were not significantly affected by NSC745885 treatment (Fig. 4A). The global histone H3 K27 trimethylation, modified by EZH2, was also not reduced by NSC745885 (Supplemental Fig. 5).

The EZH2 downstream targeted genes were also investigated to determine if they were affected by NSC745885 treatment. This resulted in the up-regulation of EZH2-silenced tumor suppressor genes, including CDKN1C, DAB2IP, and WNT5a, in a dose-dependent manner in multiple cancer cell lines (e.g., T24, MBT2, and PC3) (Figs. 4B-4D). Consistent with results that NSC745885 did not inhibit the cell growth of SV-HUC-1 immortalized normal urothelial cells, NSC745885 had no effects on EZH2 protein levels and its downstream target gene expression in SV-HUC-1 cells (Fig. 4E).

### NSC745885 down-regulated EZH2 that was responsible for its growth inhibitory effects on cancer cells

**Figure 5:**
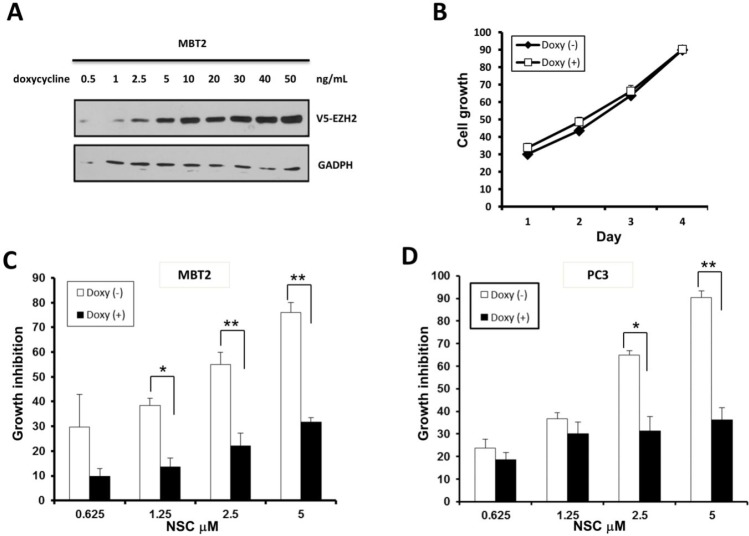
Ectopic over-expression of EZH2 attenuated the growth inhibition effect of NSC745885 (A) V5-tagged EZH2 tet-on conditional-over-expressing MBT2 stable transfectants were constructed and optimized for doxycycline induction. (B) V5-tagged EZH2 tet-on MBT2 cells were treated with or without 50 ng doxycycline. (C) V5-tagged EZH2 tet-on MBT2 cells were treated with indicated concentrations of NSC745885 in the presence or absence of 50 ng doxycycline. (D) PC3 cells transiently transfected with V5-EZH2 tet-on plasmid were treated with indicated concentrations of NSC745885 before and after doxycycline induction. All cell growth were measured by MTT assay (mean±SEM, n=3 for each group).

To investigate if EZH2 down-regulation was responsible for the growth-inhibitory effects of NSC745885 on cancer cells, TET-On, an inducible system, was used to over-express EZH2 by doxycycline in MBT2 cells. EZH2 was induced by doxycycline in a dose-dependent manner without any cytotoxic effect (Figs. 5A and 5B). This doxycycline-induced EZH2 over-expression attenuated the NSC745885-induced growth inhibition of MBT2 cells (Fig. 5C).

Whether EZH2 over-expression had any effect on NSC745885 cytotoxicity of other cancer cells was further investigated. The PC3 prostate cancer cells were transiently transfected with either vector or EZH2 plasmids, and then followed by NSC745885 treatment. The cytotoxicity of NSC745885 was also significantly attenuated in PC3 cells over-expressing EZH2 (Fig. 5D).

### NSC745885 induced the degradation of EZH2 via proteasome-mediated pathway

**Figure 6:**
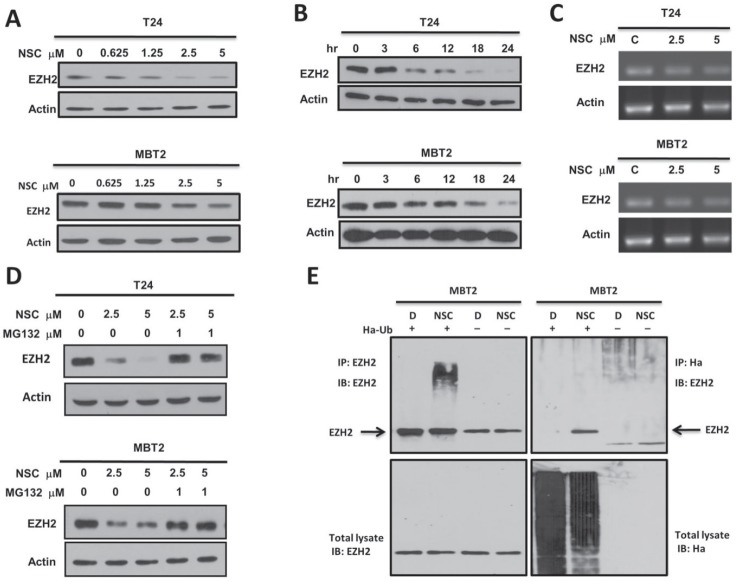
NSC745885 down-regulated EZH2 in a proteasome-mediated degradation pathway (A) T24 and MBT2 cells were treated with indicated concentrations of NSC745885. (B) T24 and MBT2 cells were treated with 5M NSC745885 in indicated time. (C) The EZH2 mRNA expression in T24 and MBT2 cells was analyzed by semi-quantitative PCR after treatment with solvent (DMSO, labeled C) or 2.5 and 5 μM NSC745885 for 24 h. Actin expression was measured as internal control. (D) T24 and MBT2 cells were treated with 2.5 and 5 μM NSC745885 alone or combined with 1 μM MG-132 for 24 h. For (A, B, and D), cells treated with DMSO were used as control and EZH2 protein levels were measured by Western blotting. (E) MBT2 cells were transiently transfected with Ha-tagged ubiquitin for 24 h. Immuno-precipitations were conducted with anti-EZH2 and anti-Ha antibodies. The precipitated samples were analyzed by immuno-blotting with antibodies against EZH2 and Ha. IgG was a non-specific pull-down control.

To investigate the molecular mechanisms involved in NSC745885-mediated EZH2 protein depletion, the dynamic effects of NSC745885 on EZH2 expression were first checked in T24 and MBT2 cancer cell lines. NSC745885 efficiently depleted EZH2 expression in a dose- and time-dependent manner (Figs. 6A and 6B). Whether NSC745885 had an effect on EZH2 mRNA expression was then investigated using real-time PCR. NSC745885 did not reduce EZH2 mRNA compared to control levels (Fig. 6C).

From these, the effect of NSC745885 on EZH2 protein stability was further examined. Treatment with the proteasome inhibitor MG132 resulted in marked suppression of NSC745885-induced EZH2 protein depletion in both T24 and MBT2 cells (Fig. 6D). EZH2 from cells treated with NSC745885 in the presence of MG-132 were further immuno-precipitated. Immuno-blotting revealed that NSC745885 treatment increased the ubiquitination of EZH2 (Fig. 6E). Taken together, these results indicated that the NSC745885-mediated down-regulation of EZH2 proteins was mainly through a proteasome-mediated ubiquitination protein degradation pathway.

### NSC745885 suppressed tumor growth and down-regulated EZH2 in xenograft animal models

**Figure 7:**
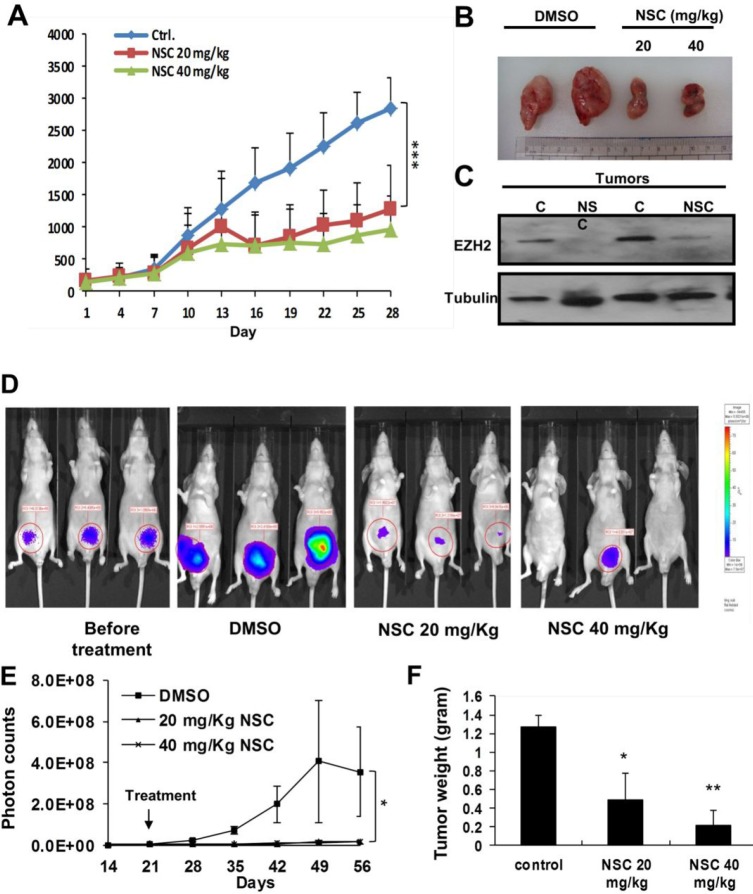
NSC745885 suppressed tumor growth and down-regulated EZH2 *in viv***o** (A) *In vivo* anti-tumor activity of NSC745885 was tested on nude mice bearing MBT2 xenografts receiving intra-peritoneal 20 and 40 mg/kg NSC745885 or DMSO every other day. The tumor volumes of mice treated with NSC745885 or DMSO were measured (n=8). ***p<0.001 (B) A representative macroscopic view of the resected xenograft tumors was shown in the indicated treatment groups. (C) EZH2 protein levels of MBT2 tumors harvested from mice treated with 3 times DMSO or NSC745885 were determined by Western blotting (C, DMSO treatment; NSC, NSC745885 treatment). (D) Bioluminescence images of mice from each treatment group were shown on the indicated day. The animals with the tumors derived from luciferase stably transfected PC-3 cells were imaged after luciferin injection using an IVIS instrument. (E) Tumor volume was analyzed by the IVIS instrument after the indicated treatments. The tumor volume of each mouse was determined by region-of-interest analysis of total photons per second (n=6 per group). (F) After 5 weeks of treatment, the tumor weights were measured, plotted, and statistically calculated as mean±SEM. *p<0.05; **p<0.01; ***p<0.001

To further investigate the *in vivo* anti-tumor activity of NSC745885, MBT2 xenografts were used as an animal model. NSC745885 showed significant anti-tumor activity in mice bearing the MBT2 xenografts at relatively low doses of 20 and 40 mg/Kg (Figs. 7A and 7B). The *in vivo* effects of NSC745885 in down-regulating EZH2 in mice bearing MBT2 tumors treated with three intra-peritoneal injections of either DMSO or 20 and 40 mg/Kg of NSC745885 were also investigated. By Western blot, fresh tumors harvested from the aforementioned experiment showed that NSC745885 efficiently down-regulated EZH2 expression *in vivo* (Fig. 7C). The body weight and daily activity of mice treated with NSC745885 did not show any significant changes compared to mice in the control group (data not shown).

Aside from using immuno-deficient mice xenografted with MBT2 tumors, the *in vivo* anti-tumor activity and safety of NSC745885 was further examined by orthotopically injecting PC3 prostate cancer cells with stable luciferase expression (PC3-Luc cells) into mice prostate. The *in vivo* luciferase activity of injected PC3-Luc cells was sequentially examined daily under the IVIS Xenogene system. Treatment with the same protocol as the xenografted experiments was started within two weeks of the injection. The photon activity and volume of tumors from mice treated with NSC745885 were significantly decreased compared to those of the control group (Figs. 7D-7F). Thus, NSC745885 had potent anti-tumor effects and down-regulated EZH2 *in vivo*.

## DISCUSSION

The present study shows that the natural product derivative NSC745885 efficiently depletes EZH2, resulting in the inhibition of cell growth in various cancer cells. Interestingly, NSC745885 has less effect on normal cells, including fibroblast and epithelial cells. The reasons why NSC745885 has selective cytotoxicity towards cancer cells need to be further investigated. Moreover, EZH2 over-expression of cancer cells attenuates the NSC745885-mediated growth inhibition, indicating that EZH2 is a major target responsible for NSC745885 pharmacologic anti-cancer activity.

Growing evidence demonstrates that histone methyltransferase EZH2 has major roles in carcinogenesis. The over-expression of EZH2 has been described in various types of cancers, including prostate, breast, bladder, gastric, lung, and hepatocellular carcinoma, which correlate with aggressive clinical manifestations [32]. The oncogenic effects of EZH2 have been experimentally demonstrated by the induction of anchorage-independent colony growth and the promotion of invasion *in vitro* by the over-expression of EZH2 in breast epithelial cells [21]. The exogenous expression of EZH2 also increases the proliferation of mouse embryonic fibroblasts [33]. A remarkable link between EZH2 binding and the aberrant methylation of CpG islands at promoters had been shown in different cancers [34-36]. The EZH2-mediated H3K27 trimethylation may act as a platform to recruit DNMTs for aberrant CpG island hyper-methylation of promoters to silence tumor suppressor genes in cancers [37].

Aside from the transcriptional repression of tumor suppressor genes, EZH2 may contribute to tumor development by misleading of cells towards a stem cell-like status. EZH2 is essential for early mammalian embryonic development [38]. Knock-out EZH2 in embryonic stem cells results in a severe defect in mesendodermal lineage commitment [39]. Supportive evidence shows that human prostate cancer cells have a gene expression signature similar to expression patterns active in embryonic stem cells [40]. A cell line study demonstrates increased EZH2 expression in CD44+/CD24- metastatic PC3-32 cells compared to cells isolated from the parent PC3 line [41].

It is interesting to know if EZH2 has a role in the “cancer stem cell” driving force behind tumor proliferation and progression [41]. Previous observations indicate that EZH2 is a perfect candidate for developing the next novel anti-cancer medicine. Since EZH2 is a histone methyltransferase, inhibition of its enzyme activity has been initially considered to be an effective way for cancer treatment. However, several kinases have been found to phosphorylate EZH2 at different sites that alter EZH2 enzyme activity. Akt and CDK1 phosphorylate EZH2 on S21 and T350, respectively, which lead to decreased methyltransferase activity but enhanced oncogenic effects [23, 24]. Post-translational modification of EZH2 also hinders its methyltransferase activity and contributes to the oncogenic processes. These clearly demonstrate that targeting the EZH2 protein itself instead of its enzyme activity will be a more efficient strategy for developing anti-cancer medicine.

The exact molecular mechanisms of how EZH2 promotes cell proliferation remain unknown. EZH2 can activate E2F-regulated genes [33], interfere with retinoic acid receptor signaling [42], and repress anti-proliferative genes [43] to facilitate cell proliferation. Depletion of EZH2 by siRNA leads to cell cycle arrest of various cancer cells at either G1 or G2 transition [18, 33, 44, 45]. A recent study has demonstrated that EZH2 represses the cell cycle regulator p27 expression in pancreatic cancer cells, resulting in G1/S arrest [43]. Another study in colon cancer cells shows that EZH2 depletion results in G1/S arrest without p27 re-expression. The findings here reveal that EZH2 depletion by NSC745885 causes G2/M cell cycle arrest in multiple cancer cell lines. Such discrepancies suggest that the spectrum of EZH2 target gene responses for cell proliferation and survival may vary in a tissue- or cell-specific manner.

Another interesting finding here is that NSC745885 overcomes multiple drug resistances with efficient EZH2 depletion of MGHU1R cancer cells. Multi-drug resistance is one of the major obstacles in cancer treatment with chemotherapeutic agents. Cancer stem cells are suggested to be a possible reason mediating multi-drug resistance by various mechanisms. The over-expression of EZH2 contributes to acquired chemoresistance in ovarian and pancreatic cancer cells [43, 46]. Furthermore, chemotherapy enhances the cancer stem cell-like population in ovarian cancer patients and cancer cell lines with high EZH2 expression. EZH2 knock-down leads to decreased ovarian cancer stem cell population and a re-acquisition of chemo-sensitivity [47].

The data in this study also demonstrate that the down-regulation of EZH2 expression also correlates with the therapeutic response to neo-adjuvant chemotherapy of patients with advanced bladder cancer. Thus, it is pivotal to further investigate the underlying mechanisms of how EZH2 participates in maintaining cancer stem cells and mediates multi-drug resistance. The present study provides evidence that NSC745885 efficiently depletes EZH2 in cancer cells, which has the advantage of overcoming the EZH2-mediated drug resistance of cancer stem cells.

The pharmacologic inhibition of EZH2 has been shown by several agents, including 3-Deazaneplanocin A (DZNep), sulforaphane, and GSK126 to show anti-cancer activities *in vitro* and *in vivo*. DZNep and sulforaphane down-regulates EZH2 through a proteasome-mediated degradation pathway [48, 49]. GSK126, a sulfonyl-methionine competitor, inhibits EZH2 enzyme activity [50]. Although these three compounds have different mechanisms of inhibiting EZH2, they all reduce the global histone H3 K27 trimethylation, which is important for maintaining the normal function of hematopoietic cells. This raises concerns that such inhibitors may exhibit important hematologic side effects [51-53].

In addition, the reduction of global H3 K27 trimethylation is associated with poor prognosis in several cancer entities [54]. Interestingly, although NSC745885 treatment results in increased ubiquitination of EZH2 that is subsequently degraded by a proteasome pathway, the global H3 K27 trimethylation is not concomitantly reduced. This phenomenon also demonstrates that NSC745885 has very low toxicity to normal cells, which is advantageous for use in the clinical setting to avoid treatment-related side effects. The ubiquitination system is a potential anti-cancer target due to different activities between normal and cancer cells. Further investigation of the detailed mechanisms of how NSC745885 differentially activates the ubiquitination system to regulate EZH2 protein stability in cancer cells is warranted.

In conclusion, this study provides *in vitro* and *in vivo* evidence that a natural product derivative, NSC745885, possesses potent anti-cancer effects. The oncogenic EZH2 is also identified as the therapeutic target of NSC745885, which has a highly selective toxicity towards cancer cells but not normal cells. The findings here also provide an ideal pre-clinical model for cancer therapeutics that warrants further investigation for clinical translation.

## METHODS

### Compound Inhibitor and Reagents

NSC745885 was synthesized and provided by Dr. Hsu-Shan Huang (Department of Pharmacy, National Defense Medical Center, ROC). The proteasome inhibitor MG132 was purchased from Calbiochem (San Diego, CA), while emodin, doxorubicin, and doxycyclined were from Sigma-Aldrich (St Louis, MO, USA). All of the drugs were dissolved in dimethylsulphoxide (DMSO) (Merck, Darmstadt, Germany).

### Plasmids and Primers

The V5-tagged EZH2 were constructed in pCDNA56-TRm-V5-EZH2, carrying both tet-on operator and tet-on repressor genes. pCDNA3.1-HA-ubiquitin was obtained from Addgene (Cambridge, MA, USA). The primers sequences used were shown in Supplementary Table2.

### Human Tissues

Human urothelial bladder tumors and matching adjacent tissues were obtained from trans-urethral resection specimens of patients before and after neo-adjuvant chemotherapy under a protocol approved by the Institutional Review Board of Tri-Service General Hospital (TSGHIRB-2-101-05-001). All of the patients provided informed consent. All of the tumor tissues used were identified as clinical high-grade and advanced urothelial cell carcinomas by the hospital's Anatomic Pathologist Board.

### Immuno-histochemistry

Tissues were fixed in formalin and embedded in paraffin for immuno-histochemistry, which was performed according to previous protocols [23]. Briefly, tissues sections were de-paraffinized in xylene and washed with serially graded ethanol. Antigen retrieval was conducted in DAKO REAL target retrieval solution (Dako) by heating at 121°C for 10 min. To quench endogenous peroxidase activity, sections were immersed in 3% H_2_O_2_ for 5 min, hybridized with primary antibodies at 4°C overnight, and visualized using the UltraVision Quanto Detection System HRP DAB kit (Thermo scientific) according to the manufacturer's protocols. The stained sections were counterstained with hematoxylin and photomicrographs were taken using an Olympus BX51 microscope.

### Cell Culture

Cells lines were obtained from the American Type Culture Collection (ATCC) or Bioresource Collection and Research Center (BCRC, Taiwan), including human breast cancer cell lines MCF-7 (MEM) and MDA-MB453 (DMEM), the human renal carcinoma cell 786-O (RPMI), human embryonic renal epithelial cell 293T (DMEM), human prostate cancer cell lines LNCaP (RPMI), PC3, and PC3-Luc (F-12), human bladder cancer cell T24 (RPMI), human urothelial cell line SV-HUC-1 (F-12), murine fibroblast NIH-3T3 (DMEM), and murine bladder cancer cell MBT2 (RPMI). Human bladder cancer MGH-U1 and its multi-drug resistant derivative, MGH-U1R, were provided by Dr. Cheng-Keng Chuang (Department of Urology, Chang Gung Memorial Hospital) [55].

All culture conditions and techniques adhered to the Bioresource Collection and Research Center (BCRC) guidelines. The culture media used, including MEM, DMEM, RPMI, and F-12, were indicated following the cell lines used. All media were prepared based on the manufacturer's recommendations and were supplemented with 10% fetal bovine serum (FBS, Biological Industries Israel Beit-Haemek Ltd., Israel), 2 mM glutamine, 1 mM sodium pyruvate, 50 U/ml of penicillin, and 50 μg/ml streptomycin. All media and supplements were purchased from Invitrogen (Grand Island, NY, USA).

### Stable Transfection of the MBT2 Cell Line

The MBT2 cell was transfected with pCDNA56-TRm-V5-EZH2 or pCDNA56-TRm-null by lipofectamin 2000 (Invitrogen, Grand Island, NY, USA). The cells were challenged with blasticidin (Invitrogen, Grand Island, NY, USA) 24 h post-transfection and this was maintained for about two weeks until single colonies clearly formed. Single colonies were picked-up, expanded, and maintained in standard DMEM medium containing blasticidin.

### Cell Viability Assay

Cell viability was determined by MTT (3- (4,5-dimethylthiazol-2-yl)-2,5-diphenyl tetrazolium bromide assay (Sigma-Aldrich, St Louis, MO, USA). Cells were seeded in a 96-well plate (Corning, NY, USA) in a density of 3,000 cells per well one day prior to the experiment. NSC745885 was mixed with culture medium in the indicated concentrations. NSC745885 treatment was performed by replacing the exhausted medium with NSC745885-containing fresh medium. The treated cells were harvested at the scheduled time points (24-, 48-, and 72-h), and incubated with 1μg/ml MTT at 37ºC for 3 h. After removing the supernatant, the deposited crystals were dissolved in 100 μl of DMSO and absorbance was measured at 560 nm using a micro-plate reader.

### Cell Cycle Analysis

The cells were seeded in 6-well plates at 3×10^5^ for 24 h and then treated with emodin or DMSO after another 24 h. The cells were then fixed with 70% ice-cold ethanol over one hour before the ethanol was removed. Subsequently, the cells were stained with PI buffer (20 μg /ml Propidium iodide, 0.1% Triton X-100, and 200 μg/ml RNase A in PBS) at 37°C for 30 min. The cell-cycle distribution was analyzed by counting the DNA content using a FACS Calibur flow cytometer.

### Western Blot Analysis

Human tissues for Western blotting were lysed in RIPA (50 mM Tris pH 7.4, 1% NP40, 150 mM NaCl, 40 mM NaF, 1 mM Na_3_VO_4_, 1 mM EDTA and 10 μl/ml of protease inhibitor cocktail) buffer. For cell lines, the cells were seeded in dishes at 60% confluence overnight and then treated with emodin or vehicle control for 24 h. The cells were lysed in ice-cold RIPA buffer according to previously described methods [56]. Protein concentrations were quantified using a BCA assay (Thermo Scientific Pierce) according to the manufacturer's protocol. Lysate 5-40 μg was loaded into each lane by Western blotting. The specific signal was detected using a secondary antibody coupled with horseradish peroxidase (Jackson ImmunoResearch Labs) and Chemilucent Plus Western Blot Enhancing Kit (Millipore, Bedford, MA, USA).

The antibodies used included anti-EZH2 (Clone: AC-22, CellSignaling, Danvers, MA, USA), anti-Actin (Sigma, St Louis, MI, USA), anti-V5 tag (AbD Serotec, USA), anti-H3K27me3 (Merck-Millipore, Upstate, Lake Placid, NY, USA), anti-EED (Abcam, Cambridge, UK), anti-Suz12 (CellSignaling, Danvers, MA, USA), anti-HA (Clone:12CA5, Roche), and anti-GAPDH (Sigma, St Louis, MI, USA). Secondary antibodies, including goat-anti-mouse-HRP and goat-anti-rabbit-HRP, were purchased from Jackson ImmunoResearch Labs (Westgrove, CA, USA).

### RNA Extraction and Real-Time PCR (RT-PCR)

Total mRNA was extracted with TRIZol Reagent (Invitrogen, Grand Island, NY, USA) following the manufacturer's instructions. The harvested RNA was quantified by a NanoDrop spectrophotomerter (Thermo Fisher Scientific Inc., Wilmington, DE, USA). Total mRNA was then converted to complementary DNA (cDNA) using a SuperScriptIII first-strand cDNA synthesis kit (Invitrogen, Grand Island, NY, USA). cDNA 2 μg were subjected to polymerase chain reaction (PCR) or real-time PCR (RT-PCR) with the primer listed in supplemental materials.

Taq DNA polymerase and reagents were purchased from Invitrogen (Grand Island, NY, USA) and real-time PCR were performed using the ABI 7500 real-time PCR system (Invitrogen-Life Technologies, ABI, Grand Island, NY, USA). Data were quantified relative to the 2 delta Ct method, based on the manufacturer's instructions.

### Immuno-precipitation

Cells treated with NSC745885 and/ or MG132 were lysed in RIPA buffer and quantified with BCA methods (Bi-cinchoninic acid, Thermo-Pierce, Rockford, IL, USA). Total protein 1 mg was prepared by incubation with protein G-sepharose (GE Amersham, UK) and subsequent incubation with specific antibodies at 4ºC for 4 h. Protein G-sepharose were added for another 12-16 h and incubated at 4ºC. After antibody and protein G-sepharose incubation, the precipitated sepharose were washed five times with RIPA buffer, and mixed and cooked with SDS loading buffer (Thermo-Fermentas, Rockfor, IL, USA) for SDS-PAGE analysis.

### Immuno-fluorescent Microscopy

Cells were seeded in poly-L-lysine-coated cover slips overnight and treated with 5 μM NSC745885 or DMSO in equal volumes for 16 h, fixed with 4% para-formaldehyde in normal saline, and counterstained with DAPI (Invitrogen, MolecularProbe, Grand Island, NY, USA). The cover slips were mounted in anti-fade Golden mounting reagent (Invitrogen, MolecularProbe, Grand Island, NY, USA). Images were taken using the Leica DM250 fluorescent microscopy system with an Andor camera (Andor Technology plc. Belfast, UK) and analyzed by the Metamorph software (Leica Microsystem, Wetzlar, D-35578, Germany). Approximately 300 EGFP-EZH2 expressing cells were counted from 10 images of each treatment. After measuring the average photon intensity, cells with oversaturated signals were eliminated in the count.

### Animal Care and Experimental Procedures

Nude mice (Balb/c nu/nu) were purchased from the National Laboratory Animal Center (NLAC, Taiwan, ROC), and housed and cared for by the Laboratory Animal Center of National Defense Medical Center. MBT2 cells 2×10^6^ were inoculated in the flank region of 6-week-old nude mice. The volume of tumor implant was monitored and NSC745885 treatment began when the average tumor volume reached approximately 100 mm^3^. NS745885 (5 uM) and vehicle (20% DMSO with 20% ethanol in Cremorph EL) in equal volumes were injected peritoneally every day for tumor volume monitoring. The Institutional Animal Care and Use Committee (IACUC) of the National Defense Animal Center approved the experimental protocols and supervised the care of animals and experimental procedures. Tumor growth rate was plotted and analyzed using the Prism graphpad 5.0, with two-tailed, paired ANOVA.

## SUPPLEMENTAL TABLES AND FIGURES


