# Supplementary material for: Pharmacologic down-regulation of EZH2 suppresses bladder cancer in vitro and in vivo
Source: Oncotarget. 2014 Mar 26;5(21):10342–55. doi: 10.18632/oncotarget.1867 (PMC4279377; doi:10.18632/oncotarget.1867)
Supplement: Supplementary file 1 [file oncotarget-05-10342-s001.pdf]

Supplemental Table 1.

Characteristics of patients with advanced bladder cancer

Case	Gender	Age	Clinical T stage	Maximal tumor size (BC)	Maximal tumor size (AC)	Size difference (%)	Chemotherapy regimen
1	F	72	2b	5	0	-100	C+G
2	M	58	3	3.8	7.44	95.8	C+G
3	M	70	3	5.5	7.15	30	C+G
4	M	78	2b	4	1.22	-69.6	Ca+G
5	M	64	2b	4.6	2.39	-48	C+G
6	M	80	4	6.5	5.28	-18.8	Ca+G
7	M	49	3	10	0.4	-96	C+G
8	F	68	2b	3.6	3.2	11.1	C+G
9	M	82	2b	7.4	1.35	-81.8	Ca+G
10	F	69	3	6	6	0	C+G
11	M	73	2b	5.6	0.53	-90.5	C+G
12	F	53	3	3.5	9.6	220	C+G

BC: before chemotherapy, AC: after chemotherapy

C: cisplatin, G: gemcitabine, Ca: carboplatin

Supplemental Table 2.

Sequence of oligonucleotide primers and probes used in Q PCR and RT–PCR

Target gene	Primer	Sequence
CDKN1C	Forward primer	5′-GGCCTCTGATCTCCGATTTCT-3′
	Reverse primer	5′-CACTTTGGGACCAGTGTACCTTCT-3′
DAB2IP	Forward primer	5′-TGGACGATGTGCTCTATGCC-3′
	Reverse primer	5′-GGATGGTGATGGTTTGGTAG-3′
WNT5A	Forward primer	5′-TAAGCCCAGGAGTTGCTTTG-3′
	Reverse primer	5′-GCAGAGAGGCTGTGCTCCTA-3′
EZH2	Forward primer	5′- TCAAAACCGCTTTCCTGG -3′
	Reverse primer	5′- TGTCCCAATGGTCAGCA -3′
GAPDH	Forward primer	5′-AACATCAAATGGGGTGAGGCC-3′
	Reverse primer	5′-GTTGTCATGGATGACCTTGGC-3′

NSC745885 down-regulated EZH2 but not EZH1 – Tong et al

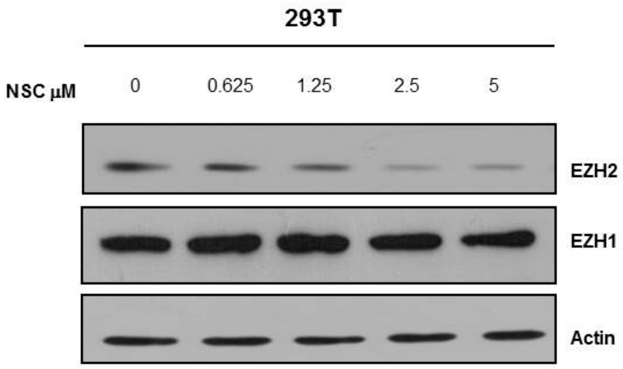

Supplemental Figure 1: 293T cells were treated with indicated concentrations of NSC745885 for 24 h. The whole cell lysates were harvested and prepared for Western blotting with the indicated antibodies.

The growth inhibition effect of NSC745885 on MBT2, T24 bladder cancer cells and SV-HUC-1 immortalized normal urothelial cells – Tong et al

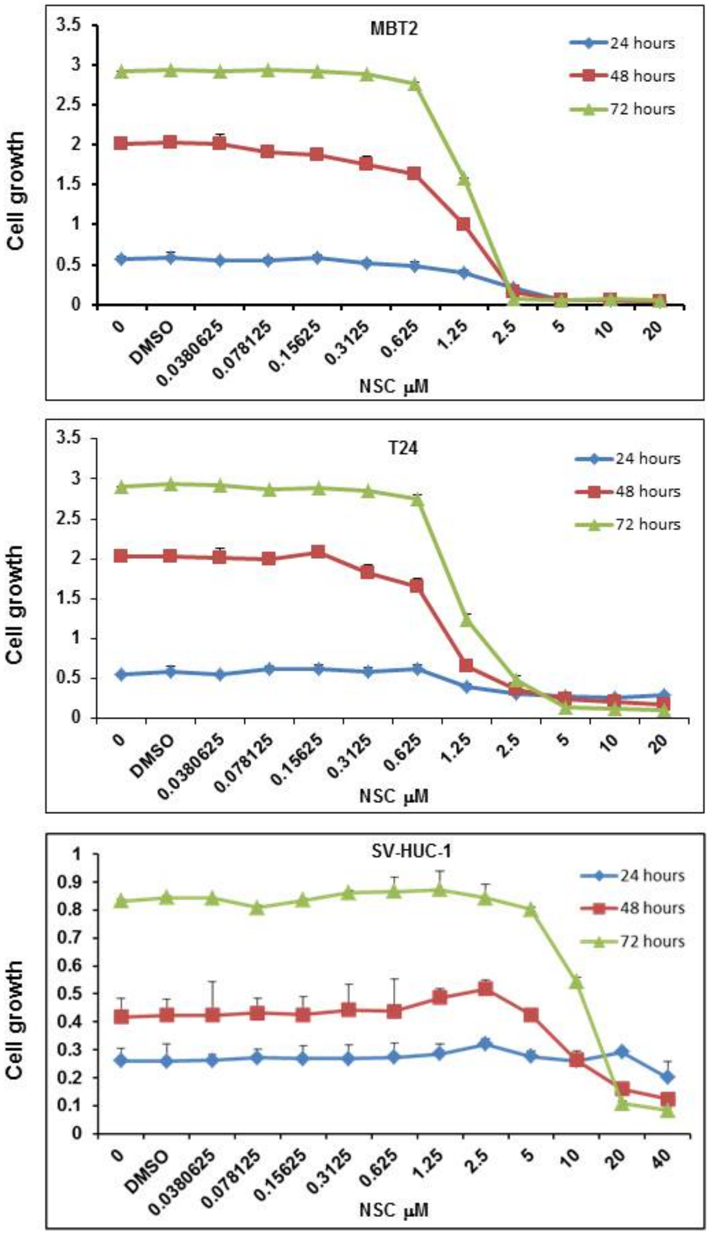

Supplemental Figure 2: MBT2, T24 bladder cancer cells and SV-HUC-1 immortalized normal urothelial cells (as indicated) were treated with indicated concentrations of NSC745885 for 24, 48 and 72 h. The growth inhibition effect of NSC745885 on MBT2, T24 bladder cancer cells and SV-HUC-1 immortalized normal urothelial cells were used MTT assay to measure cell viability at indicated times.

Serine 21 and threonine 492 phosphorylation status of EZH2 has no impact on NSC745885 down-regulation of EZH2 among bladder cancer cells and normal urothelial cells – Tong et al

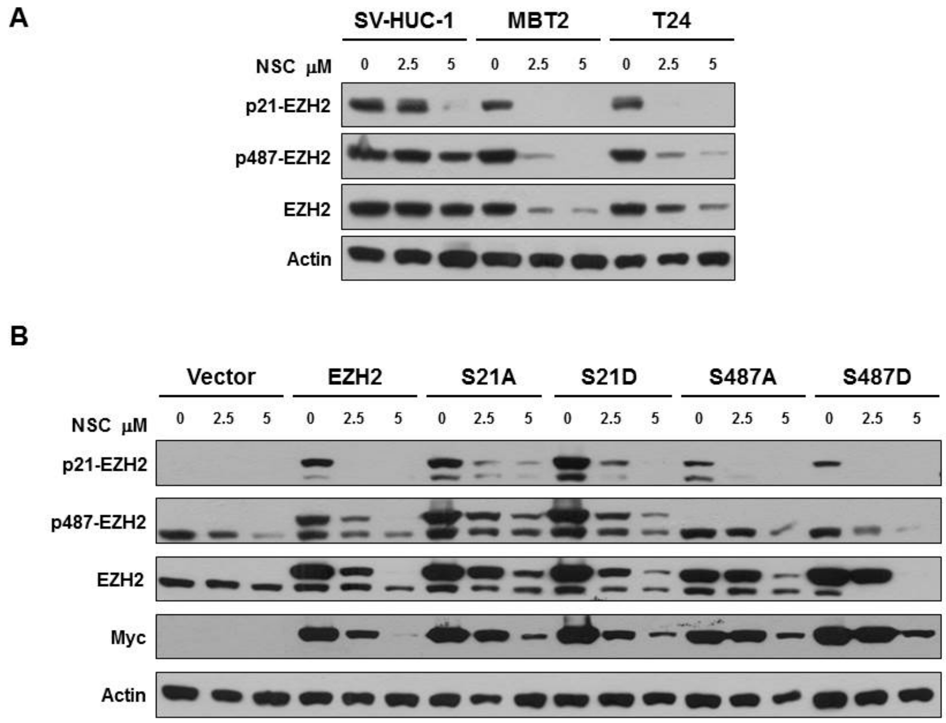

Supplemental Figure 3: A. MBT2, T24 bladder cancer cell lines and SV-HUC-1 immortalized normal urothelial cells (as indicated) were treated with indicated concentrations of NSC745885 for 24 h. The whole cell lysates were harvested and prepared for Western blotting with the indicated antibodies. B. 293T cells transiently transfected with wild-type or mutant EZH2 (21A, 21D, 492A, 492D) for 24 h then were treated with indicated concentration of NSC745885 for 24 h. Cells treated with DMSO were used as control. The whole cell lysates were harvested and prepared for Western blotting with the indicated antibodies.

NSC745885 down-regulated G2/M cell-cycle related regulators in bladder cancer cells but not in normal urothelial cells – Tong et al

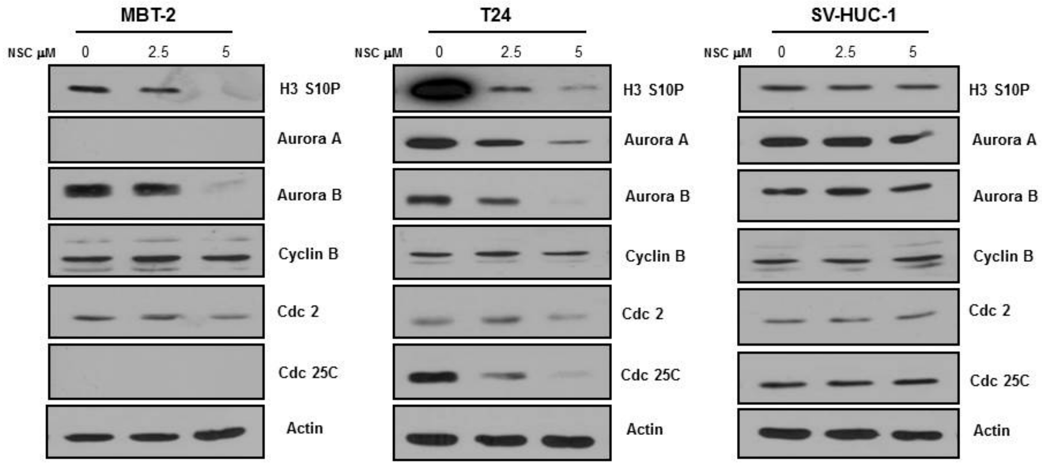

Supplemental Figure 4: MBT2, T24 bladder cancer cells and SV-HUC-1 immortalized normal urothelial cells (as indicated) were treated with indicated concentrations of NSC745885 for 24 h. The whole cell lysates were harvested and prepared for Western blotting with the indicated antibodies.

NSC745885 did not down-regulate the global H3 K27 trimethylation – Tong et al

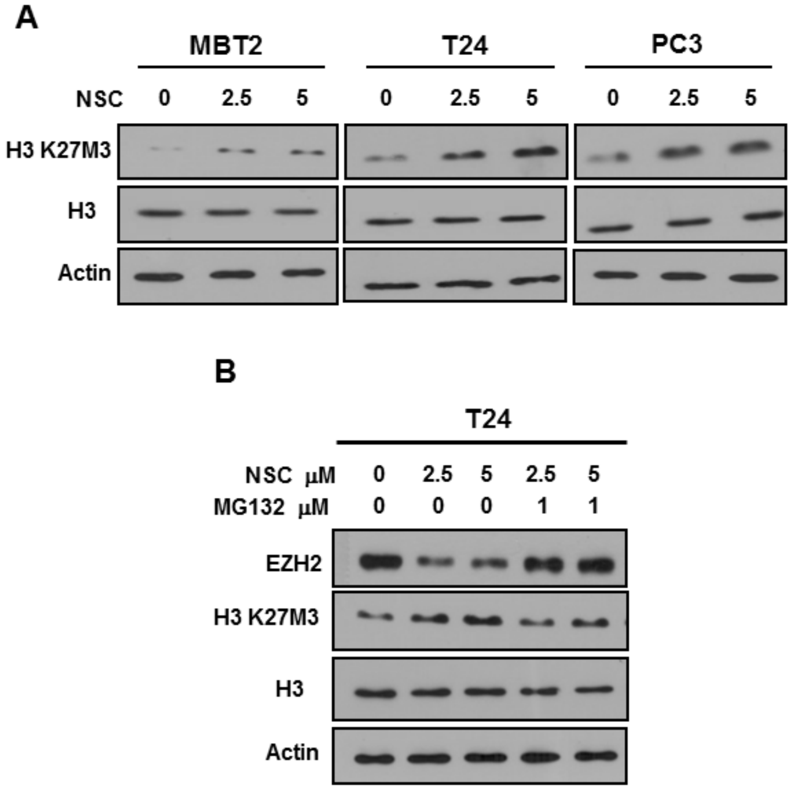

Supplemental Figure 5: A. MBT2, T24, and PC3 cancer cell lines (as indicated) were treated with indicated concentrations of NSC745885 for 24 h. The whole cell lysates were harvested and prepared for Western blotting with the indicated antibodies. B. T24 cancer cells were treated with 2.5 and 5 μM NSC745885 alone or combined with 1 μM MG-132 for 24 h. Cells treated with DMSO were used as control. The whole cell lysates were harvested and prepared for Western blotting with the indicated antibodies.
